# Multi-dimensional integration of gene expression, protein evidence, and serum autoantibodies for diagnostic modeling in esophageal squamous cell carcinoma

**DOI:** 10.3389/fimmu.2026.1707195

**Published:** 2026-02-10

**Authors:** Yuanlin Zou, Han Wang, Caijuan Song, Sirun Wang, Tiandong Li, Yifan Cheng, Hua Ye, Jianxiang Shi, Keyan Wang, Kaijuan Wang, Chunhua Song, Peng Wang, Jicun Zhu

**Affiliations:** 1 The First Affiliated Hospital of Zhengzhou University, Zhengzhou, Henan, China; 2 College of Public Health, Zhengzhou University, Zhengzhou, Henan, China; 3 Henan Key Laboratory of Tumor Epidemiology, International Joint Laboratory of Tumor Markers and Molecular Imaging, Zhengzhou University, Zhengzhou, Henan, China; 4 State Key Laboratory of Metabolic Dysregulation & Prevention and Treatment of Esophageal Cancer, Zhengzhou University, Zhengzhou, Henan, China; 5 Zhengzhou Center for Disease Control and Prevention, Zhengzhou, Henan, China; 6 Cancer Biomedicine, University College London, London, United Kingdom; 7 Prenatal Diagnosis Center, The Third Affiliated Hospital of Zhengzhou University/Maternal and Child Health Hospital of Henan Province, Zhengzhou, Henan, China; 8 Henan Institute of Medical and Pharmaceutical Sciences, Zhengzhou University, Zhengzhou, Henan, China

**Keywords:** diagnostic model, ESCC, machine learning, support vector machine, tumor-associated autoantibodies

## Abstract

**Introduction:**

Esophageal squamous cell carcinoma (ESCC) accounts for most esophageal cancer cases. This study implemented a multi-dimensional integrative approach to identify tumor-associated autoantibodies (TAAbs) and develop a diagnostic model for the early detection of ESCC.

**Methods:**

The study comprised four phases: discovery, verification, modeling, and evaluation. Transcriptomic screening of public datasets and protein-level evidence from literature were integrated to identify candidate tumor-associated antigens (TAAs), followed by serological evaluation using enzyme-linked immunosorbent assay (ELISA) in 940 samples. Eight machine learning algorithms were assessed to develop the optimal diagnostic model.

**Results:**

In the discovery phase, transcriptomic analysis identified 26 differentially expressed genes in ESCC, of which ten genes encoding proteins with literature-supported evidence were selected as candidate TAAs for serological testing. Seven TAAbs were significantly elevated in ESCC cases compared with normal controls in the verification phase. In the modeling phase, six TAAbs (anti-CEP55, anti-CKS1B, anti-ECT2, anti-KIF2C, anti-SURV, and anti-TPX2) remained elevated in ESCC cases compared with both benign esophageal disease and normal controls. The support vector machine (SVM) model demonstrated the best diagnostic performance, achieving AUCs of 0.826 (95% CI: 0.776–0.876) in the training set and 0.741 (95% CI: 0.651–0.832) in the internal test set. In the evaluation phase, the SVM model was validated in an independent temporal test set (AUC 0.779, 95% CI 0.717–0.842). The web-based diagnostic tool is accessible at https://linzou.shinyapps.io/ESCC_SVM_Model/.

**Conclusion:**

This multi-dimensional approach linking transcriptomic evidence, protein-level validation, and immunodiagnostic markers facilitated the development of a diagnostic model, which may hold promise for early detection of ESCC.

## 1 Introduction

Esophageal cancer is a malignant tumor originating in the epithelium of the esophagus. According to GLOBOCAN 2022, esophageal cancer ranks as the 11th most common cancer worldwide, with approximately 511,000 new cases, and the 7th leading cause of cancer-related deaths, accounting for about 445,000 deaths (1). East Asia exhibits the greatest incidence rates (1). In China, the burden of esophageal cancer is particularly significant, contributing to 58.50% of new cases and 56.80% of deaths globally in 2022 (2). Approximately 90% of esophageal cancer cases are classified as esophageal squamous cell carcinoma (ESCC), the most common histologic type (3). Despite the poor prognosis of esophageal cancer, with a 5-year survival rate of less than 20%, studies have demonstrated that patients with early-stage ESCC who undergo esophagectomy had a 5-year survival rate exceeding 85% (4, 5). However, early detection and treatment remain challenging due to the lack of specific clinical symptoms. Furthermore, considering the high cost and invasiveness of endoscopy and biopsy, there is a critical need for new non-invasive diagnostic biomarkers to enable earlier detection. Currently, no effective marker exists for the early diagnosis of ESCC.

The immune system detects aberrant tumor-associated antigens (TAAs) in cancer patients, triggering the production of tumor-associated autoantibodies (TAAbs) (6). Due to their long half-life, reliable detection methods, persistence, and appearance in the early stages of disease, TAAbs have been recognized as valuable biomarkers for early cancer screening (7, 8). According to Oshima Y et al., autoantibodies against NY-ESO-1, were detected in the serum of patients with various cancers, with the highest positivity rate found in esophageal cancer, suggesting that this antibody could serve as a specific biomarker for its diagnosis (7). Anti-PDLIM1 was highly expressed in ovarian cancer and had been suggested as a complementary indicator to CA125 to improve the detection of ovarian cancer (8). Due to the relatively low sensitivity of individual TAAbs, recent research has focused on combining multiple markers to achieve higher diagnostic value. This approach has already shown promise in the diagnosis of breast cancer (9), lung cancer (10), hepatocellular carcinoma (11), oral cancer (12), pancreatic adenocarcinoma (13), and other cancers. Previous studies on autoantibodies in ESCC primarily used custom protein arrays developed by researchers or serological proteome analysis to screen for potential autoantibodies (14, 15). In recent years, bioinformatics has become an effective tool for screening differentially expressed genes (DEGs) from public databases, offering a novel approach to identifying biomarkers. Protein levels are closely related to changes in mRNA levels (16). Since cellular proteins released from tumor tissues can activate the immune system to induce autoantibody production, abnormally expressed proteins may affect the level of autoantibodies in the immune system (17). However, studies that start from gene expression level and investigate autoantibody levels through tissue protein expression are limited.

Therefore, this study utilized Gene Expression Omnibus (GEO), The Cancer Genome Atlas (TCGA), and Genotype-Tissue Expression (GTEx) database and performed weighted gene co-expression network analysis (WGCNA) to identify candidate genes linked to ESCC. A literature review was conducted to explore the expression of corresponding proteins encoded by these candidate genes in ESCC, and the proteins with abnormal expression were identified as candidate TAAs. In total, 940 serum samples were measured by enzyme-linked immunosorbent assay (ELISA). Among them, ELISA was utilized to identify TAAbs with diagnostic significance for ESCC in 728 individuals. Finally, eight machine learning models were constructed based on the TAAbs identified by ELISA, and the optimal diagnostic model was selected. The performance of this model was further evaluated in an independent temporal test set of 212 samples, highlighting its potential utility for early detection of ESCC.

## 2 Method

### 2.1 Study population

This study was designed as a hospital-based case–control study. A total of 728 serum samples were collected between October 2020 and August 2023 at a tertiary hospital in Henan Province, China, including 277 patients of ESCC, 277 normal controls (NC), and 174 patients with benign esophageal disease (BED). In addition, an independent temporal test set consisting of 106 ESCC cases and 106 NC was established using samples collected from the same hospital between September 2024 and August 2025. This study was approved by the Ethics Committee of Zhengzhou University, and written informed consent was obtained from all participants. All ESCC cases were recruited from newly diagnosed patients who had not received any antitumor treatment prior to blood collection. Normal controls were recruited from individuals undergoing routine health examinations at the same hospital during the same period and were frequency-matched to ESCC cases by age and sex. Subjects with immune system-related diseases or any history of cancer were excluded. In the verification phase, 90 ESCC cases and 90 NC were used to identify differentially expressed TAAbs. These differentially expressed TAAbs were further assessed in 187 ESCC cases, 187 NC, and 174 BED patients during the modeling phase. Additionally, the 187 ESCC cases and 187 NC were randomly divided into a training set and an internal test set in a 7:3 ratio. Eight diagnostic models were trained using the training data to identify the optimal model, which was then validated in the internal test set. The final model was further evaluated for generalizability in the independent temporal test set.

Fasting venous blood samples (5 mL) were collected from all participants and allowed to clot at room temperature. After clot formation, samples were centrifuged at 3,000 rpm for 5 min to separate the serum. The supernatant was aliquoted into 1.5-mL Eppendorf tubes and stored at −80 °C. To reduce the impact of multiple freeze-thaw cycles, the serum was aliquoted into 200 μL portions.

### 2.2 Bioinformatics and literature search for screening candidate TAAs

Gene microarray datasets containing both ESCC and normal samples were obtained from the GEO database, and intersections of DEGs from seven relevant GEO datasets were analyzed (**Supplementary Table S1**; **Supplementary Figure S1**). Among these, gene dataset GSE23400, with the largest sample size, was selected for WGCNA (**Supplementary Figure S2**). The color module from WGCNA most strongly associated with ESCC was exported to Cytoscape software, where the top 50 hub genes were identified based on their connectivity with other genes using the CytoHubba plugin. The DEGs that were also hub genes were retained, resulting in a final set of 26 common DEGs (**Supplementary Figure S3**; **Supplementary Table S2**). The 26 common genes were validated using the LIMMA method across TCGA-GTEx database (78 tumor tissues and 663 normal tissues) (**Supplementary Table S3**). To reduce potential batch effects when integrating TCGA and GTEx data, batch correction was performed using the “ComBat” algorithm from the “SVA” R package. |log_2_FC|> 1 and adjusted *P* value < 0.05 were considered statistically significant for candidate genes.

Furthermore, to evaluate the protein expression levels of the 26 candidate genes, a literature review focusing on immunohistochemical analysis was conducted. The search strategy and inclusion/exclusion criteria are provided in **Supplementary Table S4**. Up to June 30, 2024, the literature review identified that the corresponding proteins of 11 out of 26 candidate genes had been reported in 20 studies, with significantly higher protein expression levels observed in ESCC tissues (**Supplementary Table S5**). These 11 highly expressed proteins served as candidate TAAs for subsequent studies.

### 2.3 Detection of differentially expressed TAAbs by ELISA

Ten proteins (ECT2, KIF4A, SURV, AURKA, CKS1B, TPX2, CEP55, NEK2, KIF2C, MCM6) were purchased from Wuhan Huamei Bioengineering Corporation, Cloud-clone Corporation, and Sanying Corporation, except for CDKN3, which was excluded due to the inability to achieve the required purity and concentration for the experiment. All proteins were diluted to 0.250 μg/mL in coating buffer after verification of their concentration, purity, and molecular weight by SDS-PAGE, confirming the expected molecular weight and a purity greater than 85%. Ninety-six-well plates were coated with the specific antigens and incubated overnight at 4 °C. After blocking with 2% BSA for 2 hours at 37 °C, serum samples were added at a 1:100 dilution and incubated for 1 hour at 37 °C. Chromogenic solutions A and B were mixed at a 1:1 ratio and added to each well, and color development was carried out in the dark for 5–15 minutes. The reaction was stopped by adding stop solution to each well. The absorbance was measured at 450 and 620 nm, with the net optical density (OD) value calculated by subtracting the 620 nm reading from the 450 nm reading. Each plate included two blank wells and six quality control (QC) wells. The QC wells contained serum samples with high immunogenicity identified in preliminary experiments, and their OD values were used to normalize each plate. Plates with blank OD > 0.1 were repeated.

The ELISA detection of autoantibodies and subsequent data processing were performed by two researchers (Yuanlin Zou and Han Wang) who were blinded to sample status. To minimize plate-to-plate variation, serum samples from patients with ESCC and BED, as well as healthy controls, were simultaneously assayed on each ELISA plate. Within each plate, sample positions were randomly assigned to reduce potential positional effects.

### 2.4 Statistical analysis

The ESCC group required 40 to 264 cases and the control group required 58 to 215 participants (alpha = 0.05, allowable error = 0.05 to 0.10, expected sensitivity = 0.20, and expected specificity = 0.85). The sample size was calculated using PASS (version 15). The levels of individual TAAbs were compared between two groups using the Mann-Whitney U test and between multiple groups using the Kruskal-Wallis H test. The optimal cutoff value for each TAAb was determined by identifying the value that maximized the Youden index (YI) while achieving a specificity of over 85%. Diagnostic performance was evaluated by calculating the area under the receiver operating characteristic (ROC) curve (AUC), sensitivity, specificity, positive likelihood ratio (+LR), negative likelihood ratio (-LR), YI, and accuracy. The 95% confidence intervals (CI) for these indicators were determined using a bootstrap resampling method (R = 1000). Eight machine learning algorithms, including support vector machine (SVM), random forest (RF), neural network (NN), naive Bayes (NB), linear discriminant analysis (LDA), mixture discriminant analysis (MDA), flexible discriminant analysis (FDA), and logistic regression (LR), were created using the “caret” R package in the training set based on the OD values of the six TAAbs. Each model’s performance was assessed through 100 iterations of 10-fold cross-validation to ensure reliable results. For each machine learning model, the optimal cutoff value was determined by maximizing the Youden index while constraining specificity to be greater than 85%. The statistically significant differences in the AUC were calculated using the DeLong test. Subgroup analysis was conducted on gender, age, TNM stage, differentiation, site, lymphatic metastasis, and distant metastasis to assess the diagnostic stability of the optimal model. Calibration curves were generated using the “rms” package in R, based on the marginal effect and the average predicted probabilities of the selected model. Decision curve analysis (DCA) was performed using the “dcurves” package to evaluate the net clinical benefit and the discriminative performance of the selected model. A web-based diagnostic tool was developed using the “shiny” package and deployed on the open-source platform “shinyapps.io” for convenient access. All statistical analyses were performed by R software (version 4.2.2), and a two-tailed *P* < 0.05 was considered statistically significant.

## 3 Result

### 3.1 Study design and demographic characteristics

**Figure 1:**
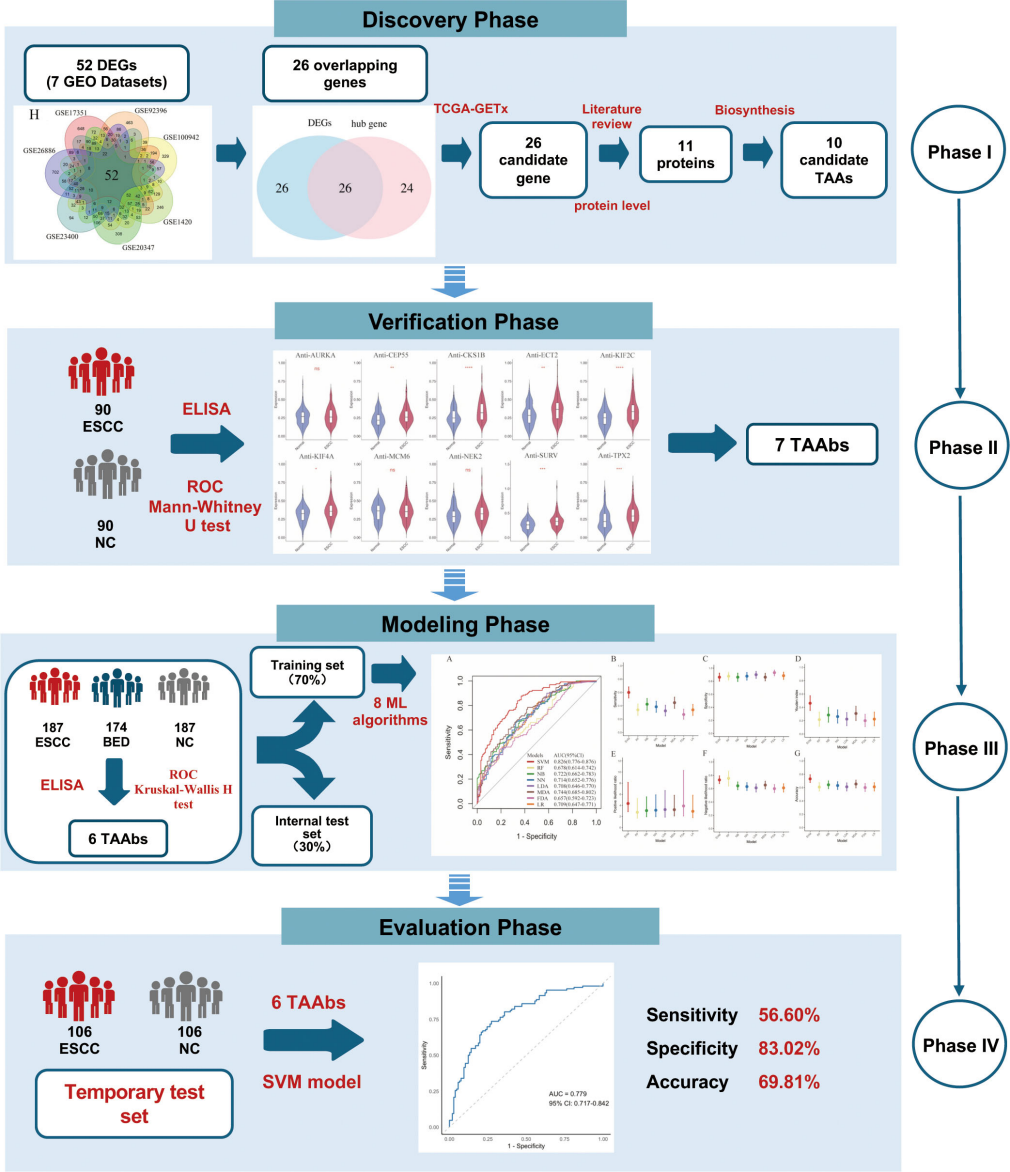
The flow diagram of this study. GEO, gene expression omnibus; DEGs, differentially expressed genes; ESCC, esophageal squamous cell carcinoma; NC, normal controls; ELISA, enzyme-linked immunosorbent assay; BED, benign esophageal disease; ML, machine learning; TAAs, tumor-associated antigens; TAAbs, tumor-associated autoantibodies; SVM, support vector machine.

The study comprised four phases: the discovery phase (Phase I), the verification phase (Phase II), the modeling phase (Phase III), and the evaluation phase (Phase IV) (**Figure 1**). In Phase I, candidate genes were identified through bioinformatics analysis, and the corresponding proteins were reviewed through a literature review to assess their expression levels. In Phase II, the levels of autoantibodies corresponding to the 10 candidate TAAs were examined in 90 ESCC cases and 90 NC. In Phase III, the levels of 7 differentially expressed TAAbs were further compared in 187 ESCC cases, 174 BED controls, and 187 NC, and six TAAbs were found to be differentially expressed between the ESCC and normal groups. These six TAAbs were then used to construct eight machine learning models. The optimal model was selected from the training set and validated in the internal test set. In Phase IV, the diagnostic performance of the optimal model was further evaluated in an independent temporal test set of 106 ESCC cases and 106 NC. Detailed demographic and clinical information for the participants is provided in **Table 1**.

**Table 1:** Characteristics of participants in the verification, modeling, and evaluation phases.

Variables	Verification phase		modeling phase				Evaluation phase	
	Verification set		Training set		Internal test set		Temporal test set	
	ESCC	NC	ESCC	NC	ESCC	NC	ESCC	NC
N	90	90	130	130	57	57	106	106
Age (median,IQR)	69.50 (10.00)	69.50 (10.00)	66.50 (12.00)	67.00 (10.00)	68.00 (12.00)	68.00 (8.00)	69.00 (13.00)	68.50 (14.00)
Age (mean,SD)	67.94 (8.61)	67.98 (8.65)	66.34 (8.84)	66.87 (8.41)	67.88 (8.55)	67.79 (7.48)	68.03 (9.80)	67.93 (9.97)
Gender, n (%)								
Male	63 (70.00)	63 (70.00)	94 (72.30)	90 (69.23)	36 (63.16)	40 (70.18)	76 (71.70)	76 (71.70)
Female	27 (30.00)	27 (30.00)	36 (27.69)	40 (30.77)	21 (36.84)	17 (29.82)	30 (28.30)	30 (28.30)
Site, n (%)								
Upper	9 (10.00)		12 (9.23)		10 (17.54)		13 (12.26)	
Middle	35 (38.89)		50 (38.46)		21 (38.84)		42 (39.62)	
Lower	11 (12.22)		18 (13.85)		11 (19.30)		12 (11.32)	
Unknown	35 (38.89)		50 (38.46)		15 (28.32)		39 (36.79)	
Differentiation, n (%)								
Low	9 (10.00)		10 (7.69)		3 (5.26)		12 (11.32)	
Moderate	14 (15.56)		23 (17.69)		15 (26.32)		21 (19.81)	
High	17 (18.89)		29 (22.31)		14 (24.56)		25 (23.58)	
Unknown	50 (55.56)		68 (52.31)		25 (43.86)		48 (45.28)	
TNM stage, n (%)								
I-II	26 (28.89)		41 (31.54)		24 (42.11)		29 (27.36)	
III-IV	19 (21.11)		19 (14.62)		10 (17.54)		20 (18.87)	
Unknown	45 (50.0)		70 (53.85)		23 (40.35)		57 (53.77)	
Lymphatic metastasis, n (%)								
Yes	22 (24.44)		28 (21.54)		15 (26.32)		24 (22.64)	
No	20 (22.22)		39 (30.00)		21 (36.84)		29 (27.36)	
Unknow	48 (53.33)		63 (48.46)		21 (36.84)		53 (50.00)	
Distant metastasis, n (%)								
Yes	7 (7.78)		8 (6.15)		7 (12.28)		5 (4.72)	
No	28 (31.11)		49 (37.69)		23 (40.35)		38 (35.85)	
Unknown	55 (61.11)		73 (56.15)		27 (47.37)		63 (59.43)	

ESCC, Esophageal squamous cell carcinoma; NC, Normal controls.

### 3.2 Identification of candidate TAAs based on bioinformatics and literature review

A total of 52 genes were consistently identified as differentially expressed across seven GEO datasets when comparing ESCC and normal tissues (**Supplementary Table S1**; **Supplementary Figure S1**). Additionally, the GSE23400 dataset was selected for WGCNA (**Supplementary Figure S2**). The top 50 hub genes, ranked by degree of connectivity in the WGCNA network, were intersected with the 52 DEGs, resulting in 26 overlapping genes (**Supplementary Figure S3**; **Supplementary Table S2**). These 26 overlapping genes were further validated using the TCGA-GTEx database, confirming their differential expression between ESCC and normal tissues (**Supplementary Table S3**). These 26 overlapping genes were considered preliminary candidates. A comprehensive literature review (up to June 30, 2024) revealed that proteins corresponding to 11 of these genes (ECT2, KIF4A, CDKN3, SURV, AURKA, CKS1B, TPX2, CEP55, NEK2, KIF2C, MCM6) were reported to be significantly overexpressed in ESCC tissues compared with adjacent normal tissues, as confirmed by immunohistochemistry (**Supplementary Table S5**). Of these, ten proteins were successfully synthesized (excluding CDKN3) and selected as candidate TAAs for subsequent experimental validation of autoantibody responses.

### 3.3 Serum levels of autoantibodies in the verification phase

**Figure 2:**
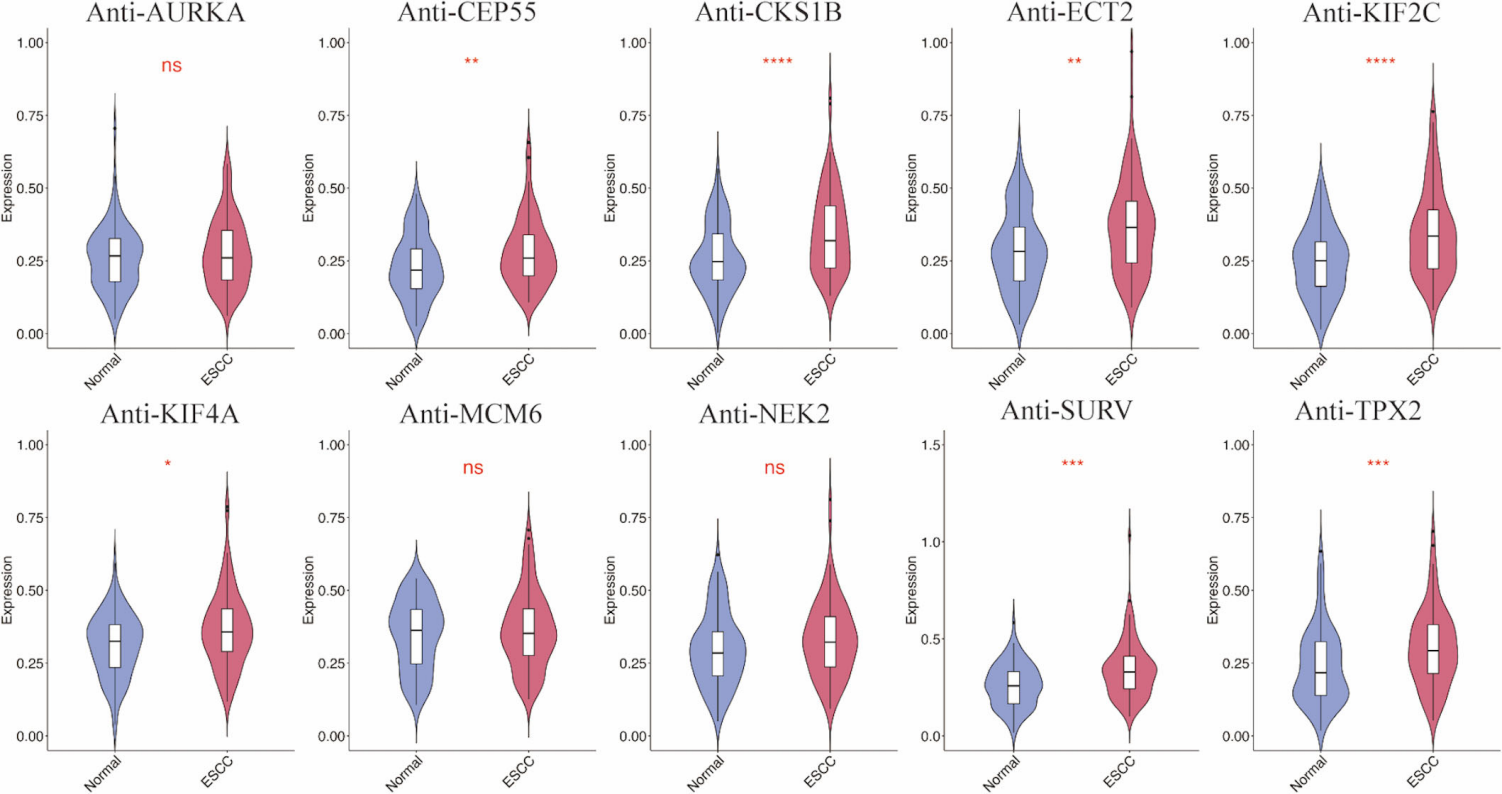
Serum levels of 10 TAAbs between 90 ESCC and 90 NC in the verification phase. ESCC, esophageal squamous cell carcinoma; NC, normal controls.

In the verification phase, ten candidate TAAs were used to assess the serum levels of TAAbs in 90 ESCC cases and 90 NC. Seven of these TAAbs (anti-CEP55, anti-CKS1B, anti-ECT2, anti-KIF2C, anti-KIF4A, anti-SURV, and anti-TPX2) were expressed at significantly higher levels in the ESCC group compared to the controls and showed potential diagnostic value (all *P* < 0.05) (**Figure 2**; **Table 2**). The AUC values for these seven TAAbs ranged from 0.607 to 0.683: anti-CEP55 (AUC: 0.615, 95% *CI*: 0.533-0.697), anti-CKS1B (AUC: 0.681, 95% *CI*: 0.604-0.758), anti-ECT2 (AUC: 0.635, 95% *CI*: 0.554-0.715), anti-KIF2C (AUC: 0.683, 95% *CI*: 0.606-0.760), anti-KIF4A (AUC: 0.607, 95% *CI*: 0.525-0.689), anti-SURV (AUC: 0.657, 95% *CI*: 0.578-0.736), and anti-TPX2 (AUC: 0.644, 95% *CI*: 0.563-0.724). Sensitivity ranged from 16.67% to 37.78%, while specificity varied from 85.56% to 95.56%.

**Table 2:** Diagnostic performance of the 10 TAAbs in the verification phase.

TAAbs	AUC (95%*CI*)	*P*	Sen (%)	Spe (%)	YI	+LR	-LR	Acc (%)
AURKA	0.535 (0.451-0.620)	0.413	22.22	88.99	0.11	2.02	0.87	52.67
CEP55	0.615 (0.533-0.697)	0.006	16.67	92.22	0.09	2.14	0.90	52.14
CKS1B	0.681 (0.604-0.758)	<0.001	34.44	85.56	0.20	2.39	0.77	54.81
ECT2	0.635 (0.554-0.715)	0.001	16.67	95.56	0.12	3.75	0.87	52.94
KIF2C	0.683 (0.606-0.760)	<0.001	37.78	85.56	0.23	2.62	0.73	55.61
KIF4A	0.607 (0.525-0.689)	0.011	22.22	94.44	0.17	4.00	0.82	54.01
MCM6	0.515 (0.430-0.600)	0.731	11.11	98.89	0.10	10.01	0.90	52.41
NEK2	0.574 (0.490-0.657)	0.084	6.67	96.67	0.03	2.00	0.97	50.80
SURV	0.657 (0.578-0.736)	<0.001	27.78	91.11	0.19	3.12	0.79	54.55
TPX2	0.644 (0.563-0.724)	<0.001	34.44	85.56	0.20	2.39	0.77	54.81

AUC, area under the ROC curve; *CI*, confidence interval; Sen, sensitivity; Spe, specificity; Acc, Accuracy; YI, Youden index; +LR, Positive likelihood ratio; -LR, Negative likelihood ratio.

### 3.4 Diagnostic performance in the modeling phase

**Figure 3:**
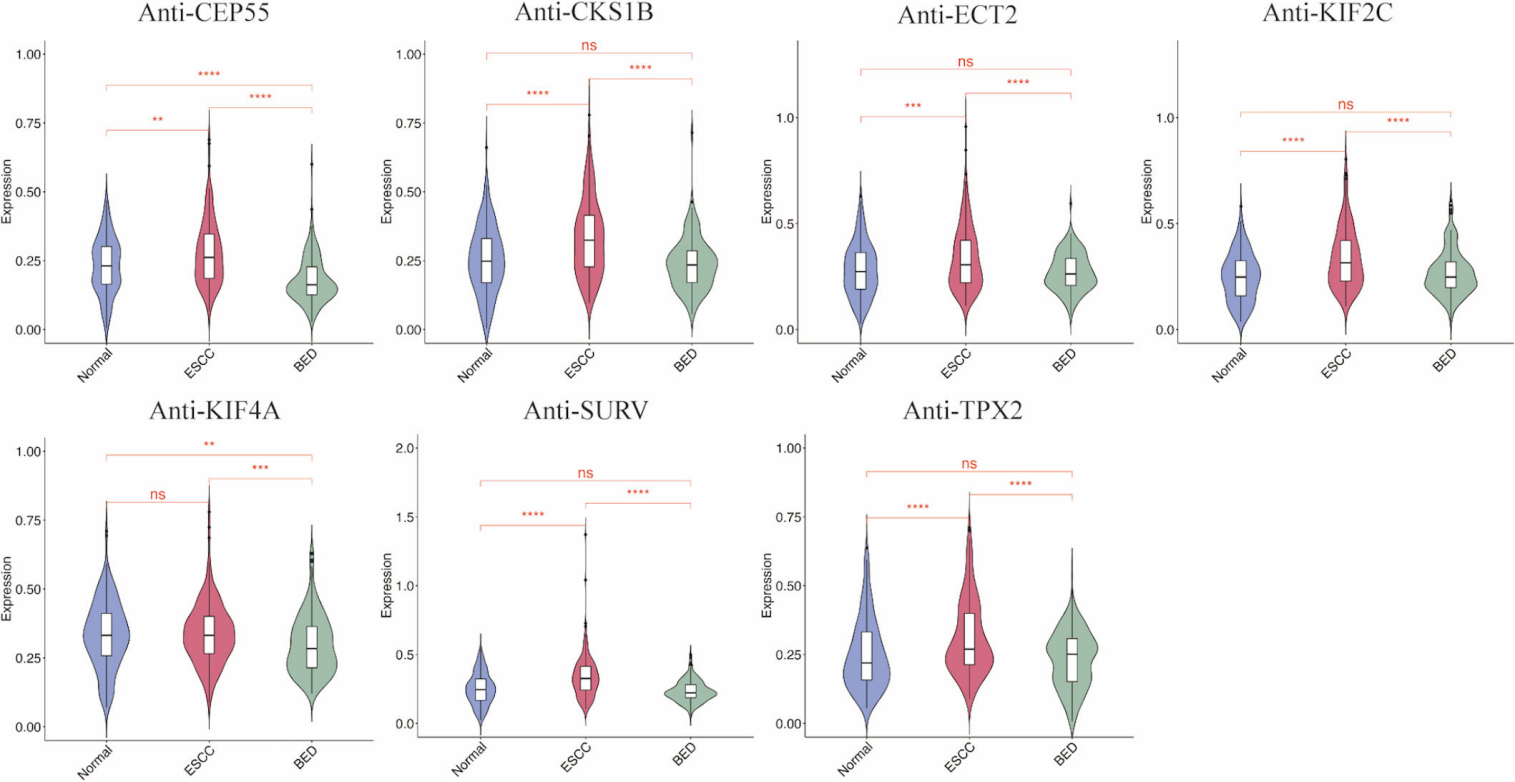
Serum levels of 7 TAAbs in 187 ESCC, 174 BED, and 187 NC in the modeling phase. ESCC esophageal squamous cell carcinoma, BED benign esophageal disease; NC normal controls.

In the modeling phase, serum levels of seven differentially expressed TAAbs from the verification phase were tested in 548 subjects, including 187 ESCC cases, 174 BED patients, and 187 NC. With the exception of anti-KIF4A, the levels of the other six TAAbs were significantly higher in ESCC group than in both BED and normal group and had diagnostic value (all *P* < 0.01) (**Figure 3**; **Table 3**). The AUC values for these six TAAbs ranged from 0.598 to 0.674: anti-CEP55 (AUC: 0.598, 95% *CI*: 0.541-0.655), anti-CKS1B (AUC: 0.657, 95% *CI*: 0.602-0.711), anti-ECT2 (AUC: 0.600, 95% *CI*: 0.543-0.657), anti-KIF2C (AUC: 0.674, 95% *CI*: 0.621-0.728), anti-SURV (AUC: 0.673, 95% *CI*: 0.619-0.727), and anti-TPX2 (AUC: 0.637, 95% *CI*: 0.581-0.693). Sensitivity ranged from 20.32% to 29.41%, and specificity varied from 85.56% to 91.98%.

**Table 3:** Diagnostic performance of the 7 TAAbs in the modeling phase.

TAAbs	AUC (95%*CI*)	*P*	Sen (%)	Spe (%)	YI	+LR	-LR	Acc (%)
CEP55	0.598 (0.541-0.655)	0.001	20.32	91.44	0.12	2.37	0.87	55.88
CKS1B	0.657 (0.602-0.711)	<0.001	28.34	89.30	0.18	2.65	0.80	58.82
ECT2	0.600 (0.543-0.657)	0.001	27.27	86.10	0.13	1.96	0.84	56.68
KIF2C	0.674 (0.621-0.728)	<0.001	28.34	91.98	0.20	3.53	0.78	62.83
KIF4A	0.500 (0.442-0.669)	0.992	2.67	98.40	0.01	1.67	0.99	50.53
SURV	0.673 (0.619-0.727)	<0.001	25.67	91.44	0.17	3.00	0.81	58.56
TPX2	0.637 (0.581-0.693)	<0.001	29.41	85.56	0.15	2.04	0.83	57.49

AUC, area under the ROC curve; CI, confidence interval; Sen, sensitivity; Spe, specificity; Acc, Accuracy; YI, Youden index; +LR, positive likelihood ratio; -LR, negative likelihood ratio.

### 3.5 Diagnostic performance of the immunodiagnostic model based on machine learning

**Figure 4:**
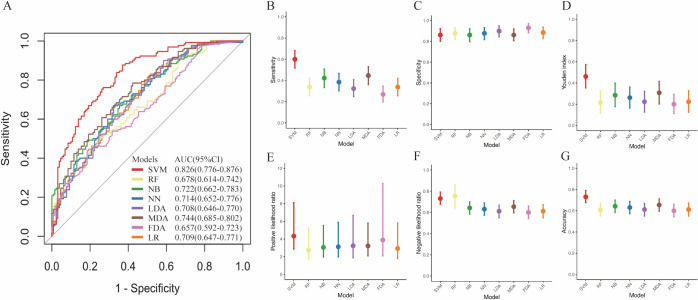
Diagnostic performance of 8 machine learning models in training set. ROC curve **(A)**, sensitivity **(B)**, specificity **(C)**, Youden index **(D)**, positive likelihood ratio (+LR) **(E)**, negative likelihood ratio (-LR) **(F)**, and accuracy **(G)** for the 8 models. SVM, support vector machine; RF, random forest; NN, neural network; NB, naive Bayes; LDA, linear discriminant analysis; MDA, mixture discriminant analysis; FDA, flexible discriminant analysis; LR, logistic regression; AUC, area under the ROC curve; CI, confidence interval.

**Figure 5:**
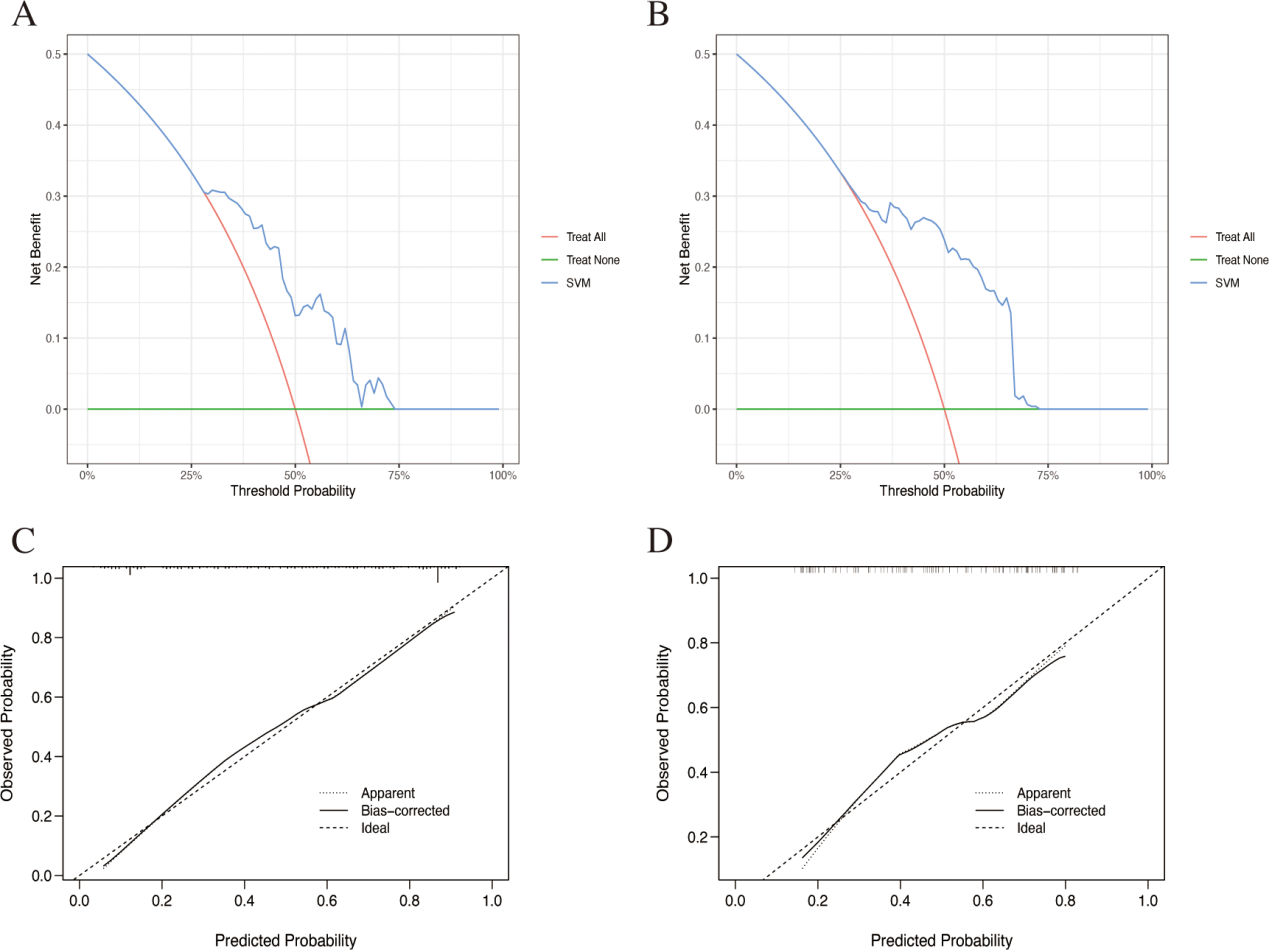
Clinical decision curves and calibration curves of SVM model in training set **(A, C)**, and internal test set **(B, D)**.

Eight machine learning models were constructed based on six TAAbs in the training set. The results of the Delong test demonstrated that the diagnostic performance of the SVM model was superior to that of the other models (**Table 4**, **Figure 4**). The AUC of the SVM model was 0.826 (95% *CI*: 0.776-0.876), with sensitivity, specificity, and accuracy rates of 60.0%, 86.15%, and 73.08%, respectively. The SVM model demonstrated an AUC of 0.741 (95% *CI*: 0.651-0.832), with a sensitivity of 45.61%, specificity of 85.96%, accuracy of 65.79% in the internal test set (**Table 5**). Although the AUC was slightly lower than in the training set, the difference was not statistically significant by DeLong test (*P* = 0.109). The DCA indicated that the SVM model provided a positive net clinical benefit compared with both the treat-all and treat-none strategies within a threshold probability range of approximately 25%–75% (**Figures 5A, B**). Calibration curves in both the training and internal test sets showed good agreement between predicted and observed probabilities (**Figures 5C, D**).

**Table 4:** Diagnostic performance of the 8 machine learning algorithms in the training set.

Evaluation index	SVM	RF	NB	NN	LDA	MDA	FDA	LR
AUC	0.826	0.678	0.722	0.714	0.708	0.744	0.657	0.709
Sen (%)	60.00	33.85	42.31	38.46	32.31	44.62	26.92	33.85
Spe (%)	86.15	87.69	86.15	87.69	90.00	86.15	93.08	88.46
YI	0.46	0.22	0.28	0.26	0.22	0.31	0.20	0.22
+LR	4.33	2.75	3.06	3.13	3.23	3.22	3.89	2.93
-LR	0.46	0.75	0.67	0.70	0.75	0.64	0.79	0.75
Acc (%)	73.08	60.77	64.23	63.08	61.15	65.38	60.00	61.15
*P* for Delong	—	<0.001	<0.001	<0.001	<0.001	<0.001	<0.001	<0.001

SVM, support vector machine; RF, random forest; NN, neural network; NB, naive Bayes; LDA, linear discriminant analysis; MDA, mixture Discriminant Analysis; FDA, flexible discriminant analysis; LR, logistic regression; AUC, area under the ROC curve; CI, confidence interval; Sen, sensitivity; Spe, specificity; Acc, Accuracy; YI, Youden index; +LR, positive likelihood ratio; -LR, negative likelihood ratio.

**Table 5:** Diagnostic performance of the support vector machine model in the internal and temporal test sets.

Group	AUC (95% *CI*)	Sen (%)	Spe (%)	YI	+LR	-LR	Acc (%)
Internal test set	0.741 (0.651-0.832)	45.61	85.96	0.32	3.25	0.63	65.79
Temporal test set	0.779 (0.717-0.842)	56.60	83.02	0.40	3.33	0.52	69.81

AUC, area under the ROC curve; 95% CI, 95% confidence interval; Sen, sensitivity; Spe, specificity; Acc, Accuracy; YI, Youden index; +LR, positive likelihood ratio; -LR, negative likelihood ratio.

### 3.6 Performance of the SVM model in the independent temporal test set

The SVM model achieved an AUC of 0.779 (95% CI: 0.717–0.842), with a sensitivity of 56.60%, specificity of 83.02%, and accuracy of 69.81% in the independent temporal test set (**Table 5**). These results indicate that the model maintained stable diagnostic performance when applied to temporally independent samples.

### 3.7 Subgroup analysis using the SVM model

In the subgroup analysis, 187 ESCC cases and 187 controls were categorized into different subgroups according to age, gender, TNM stage, differentiation, site, lymphatic metastasis, and distant metastasis. The AUC values of the SVM model ranged from 0.732 to 0.831 across the clinical subgroups. The DeLong test revealed no statistically significant differences between the subgroups (*P* > 0.05) (**Supplementary Figure S4**; **Supplementary Table S6**).

### 3.8 Application of SVM model

To enhance clinical applicability, the SVM model, identified as the best-performing classifier, was implemented as an interactive web-based diagnostic tool. This platform allows users to input the expression levels of six TAAbs (anti-CEP55, anti-CKS1B, anti-ECT2, anti-KIF2C, anti-SURV, and anti-TPX2) to generate individualized ESCC risk predictions. The web application is freely accessible at https://linzou.shinyapps.io/ESCC_SVM_Model/.

## 4 Discussion

In the current study, 11 potential biomarkers for the early diagnosis of ESCC were selected through bioinformatics analysis and a literature review. After validation using ELISA, six TAAbs (anti-CEP55, anti-CKS1B, anti-ECT2, anti-KIF2C, anti-SURV, and anti-TPX2) were identified as potential early diagnostic markers for ESCC. Eight machine learning models based on serum TAAbs were developed for early ESCC diagnosis, with the SVM model demonstrating the best diagnostic performance (DeLong *P* < 0.05), achieving an AUC of 0.826 (95%*CI* 0.776-0.876), sensitivity of 60.00%, and specificity of 86.15%. The model showed satisfactory stability in the internal test set (DeLong *P* = 0.109), with an AUC of 0.741(95%*CI* 0.651-0.832), sensitivity of 45.61% and specificity of 85.96%. Importantly, the diagnostic performance of the SVM model was further confirmed in an independent temporal test set, achieving an AUC of 0.779 (95% CI: 0.717–0.842), sensitivity of 56.60%, and specificity of 83.02%, indicating that the model maintained robust predictive ability in temporally independent samples. Subgroup analysis also indicated that the SVM model exhibited satisfactory diagnostic performance across different clinical subgroups, including age, gender, TNM stage, differentiation, site, lymphatic metastasis, and distant metastasis.

There are several methods to screen for TAAbs, with serum proteomic analysis and protein microarrays being the most common. Klade et al. compared the proteomics of non-tumorigenic kidneys and renal cell cancer by two-dimensional gel electrophoresis and silver staining to screen for potential renal cell carcinoma-associated autoantibodies (18). As early as 2009, Kijanka proposed that protein array technology could serve as a high-throughput technique capable of evaluating humoral immune reactions against thousands of proteins in cancer, thus facilitating the identification of new serum autoantibodies (19). In recent years, novel methods for screening tumor-associated TAAbs have emerged (14). For example, Chen et al. utilized serological proteome analysis combined with nanoliter-liquid chromatography and quadrupole time of flight tandem mass spectrometry for preliminary screening of ESCC-associated TAAbs (14). More recently, Xu et al. carried out a multicenter retrospective study in which serological proteome analysis was employed to identify TAAs and to evaluate the corresponding serum autoantibodies (20). Additionally, some scholars have identified oral cancer-related DEGs by public databases, and the proteins encoded by these genes as potential biomarkers (12). However, it is unclear whether this approach, which uses DEGs as a starting point for identifying ESCC-associated TAAbs, is highly effective for the early diagnosis of ESCC. In the present study, we utilized seven GEO databases, related to ESCC to identify DEGs, and performed WGCNA to select 26 candidate genes, which were further validated using data from the TCGA and GTEx databases. Eleven of the proteins encoded by these 26 candidate genes have been reported in the literature, all of which are highly expressed in ESCC. These proteins were then used as TAAs, which were further screened by ELISA to identify TAAbs for model construction. The AUC values of individual TAAbs identified by this method were almost always greater than 0.600. Our study integrated bioinformatics analysis, literature review, and experimental validation to identify ESCC-associated autoantibodies, progressing from the mRNA level to the protein level. This multi-dimensional approach highlights the value of combining transcriptomic, proteomic, and serological evidence to improve the identification of reliable diagnostic biomarkers. This strategy may provide new insights into the discovery of early diagnostic markers for other types of cancer.

Nowadays, research on autoantibodies has expanded beyond the diagnostic value of individual autoantibody, with increasing focus on combining multiple autoantibodies to construct diagnostic models, especially in cancer (10, 11, 13, 21). Jiang et al. developed a logistic regression model incorporating individual characteristics and four TAAbs (anti-GAGE7, anti-MAGE-A1, anti-CA125, and anti-CEA) to increase the precision of lung cancer detection (10). Similarly, combining three autoantibodies (anti-ZIC2, anti-CDC37L1, and anti-DUSP6) with alpha-fetoprotein significantly improved the positive diagnostic rate for early hepatitis B virus-related hepatocellular carcinoma, reaching 84.1% (11). Zhuang et al. used a human protein microarray to screen for TAAbs associated with pancreatic adenocarcinoma, achieving an AUC of 0.97 for a multivariate logistic model based on CA19–9 and CLDN17 (13). These studies have demonstrated that combining multiple autoantibodies can overcome the lower sensitivity limitations of single markers, enhancing overall diagnostic performance. This approach is likely effective because tumorigenesis is a complicated process with diverse etiologies, numerous genes changing, and various stages, especially in ESCC (22). The complementary actions of multiple autoantibodies contribute to higher diagnostic value. Therefore, this study constructed eight diagnostic models by combining multiple potentially diagnostic TAAbs through different machine learning algorithms to maximize the combined effect of multiple antibodies. Among the eight models constructed in the training set, all models had AUC values above 0.650. Among them, the SVM model achieved the highest AUC of 0.826, and its stability was further confirmed by the internal and temporal test set.

Among the six ELISA-validated TAAbs, anti-KIF2C demonstrated the best diagnostic performance. Throughout the cell cycle, KIF2C is found in the cytoplasm and could assists in normal spindle formation, normal chromosome segregation, and correction of aberrant microtubule-chromosome attachment (23–25). Recent studies suggest that KIF2C is closely linked to tumor progression, invasion, metastasis, and poor prognosis in various cancers (26). This may be due to KIF2C’s involvement in multiple cell signaling pathways (e.g. MEK/ERK (27), Wnt/β-catenin (28), P53 (29), TGF-β1/Smad (30) et al) and its role in regulating the tumor immune microenvironment (31) and repairing DNA damage in tumor cells (32). By regulating the RhoA-ERK signaling pathway, ECT2—another TAAb confirmed in this study—promotes the growth, migration, and invasion of ESCC (33). Zheng et al. previously included ECT2 in a protein-based diagnostic model for ESCC, though that study was limited to fewer than 90 ESCC cases (34). In our study, we used a larger sample size to demonstrate the diagnostic utility of anti-ECT2. Similarly, SURV, a protein encoded by the BIRC5 gene, promotes ESCC oncogenesis by binding to the IKKβ promoter, triggering NF-κB p65 activation and enhancing its transcription (35). According to Hsu et al., TPX2 is a key modulator of cell division and a poor prognostic marker in ESCC; its elevated expression is associated with reduced overall and disease-free survival (36). CEP55 has also been found to be associated with poor prognosis in patients with advanced ESCC and can promote proliferation, migration, and invasion of ESCC through the PI3K/Akt pathway (37, 38). CKS1B was found by Wang et al. to correlate with advanced tumor stage, positive lymph node metastasis, and increased resistance to radiotherapy in ESCC (39).

Several limitations must be considered. First, when searching for immunohistochemical evidence of proteins encoded by candidate genes, we excluded those proteins for which no relevant reports were available. This may have led to the omission of potentially diagnostically valuable TAAbs. Therefore, future research should continue to explore the diagnostic potential and biological mechanisms of autoantibodies produced by these proteins. Second, prospective studies with large sample sizes are needed to validate the predictive performance of the SVM model identified in this study.

## 5 Conclusion

In summary, this study used a multi-dimensional approach—integrating bioinformatics, literature review, and experimental validation—to develop an SVM model incorporating six autoantibodies (anti-CEP55, anti-CKS1B, anti-ECT2, anti-KIF2C, anti-SURV, and anti-TPX2) for the early diagnosis of ESCC. Future research should focus on validating the model in larger populations and further exploring the molecular mechanisms underlying the associated autoantibodies.

## Data Availability

The raw data supporting the conclusions of this article will be made available by the authors, without undue reservation.

## Supplementary material

Supplementary Figure 1 Volcano plots for differential expression analysis of the 7 GEO datasets **(A–G)** and Venn diagrams **(H)** for the intersection of differentially expressed genes.

Supplementary Figure 2 WGCNA analysis results. **(A)** Hierarchical clustering dendrogram of genes, **(B)** module-trait correlation heatmap, and **(C)** module eigengene-trait correlation heatmap.

Supplementary Figure 3 Venn Diagram of Hub Genes and DEGs. DEGs differentially expressed genes.

Supplementary Figure 4 ROC curves of the support vector machine model in various clinical subgroups. AUC area under the ROC curve; CI confidence interval.
