# Supplementary material for: Multi-dimensional integration of gene expression, protein evidence, and serum autoantibodies for diagnostic modeling in esophageal squamous cell carcinoma
Source: Front Immunol. 2026 Feb 10;17:1707195. doi: 10.3389/fimmu.2026.1707195 (PMC12929454; doi:10.3389/fimmu.2026.1707195)
Supplement: Supplementary Figure 1 — Volcano plots for differential expression analysis of the 7 GEO datasets (A–G) and Venn diagrams (H) for the intersection of differentially expressed genes. [file Table1.docx]

Table S1. Data sources and sample sizes used for candidate gene analysis

| Datasets | ESCC (n) | Normal samples (n) |
| --- | --- | --- |
| GEO datasets |  |  |
| GSE17351 | 5 | 5 |
| GSE92396 | 10 | 9 |
| GSE100942 | 4 | 4 |
| GSE1420 | 8 | 16 |
| GSE20347 | 17 | 17 |
| GSE23400 | 53 | 53 |
| GSE26886 | 9 | 39 |
| TCGA dataset | 78 | 11 |
| GTEx dataset | 0 | 652 |

Table S2. The 26 candidate genes and corresponding proteins

| Gene |  | Encoded protein |
| --- | --- | --- |
| ASPM | Abnormal spindle-like microcephaly associated | ASPM |
| MCM2 | Minichromosome maintenance protein 2 | MCM2 |
| MCM6 | Minichromosome maintenance protein 6 | MCM6 |
| AURKA | Aurora kinase A | AURKA |
| TPX2 | Targeting protein for Xenopus kinesin-like protein 2 | TPX2 |
| BIRC5 | Baculoviral IAP repeat-containing 5 | SURV |
| KIF4A | Kinesin family member 4A | KIF4A |
| CDKN3 | Cyclin-dependent kinase inhibitor 3 | CDKN3 |
| RAD51AP1 | RAD51 associated protein 1 | RAD51AP1 |
| GMNN | Geminin | GMNN |
| TTK | Thymoma viral proto-oncogene 1 | TTK |
| ECT2 | Epithelial cell transforming 2 | ECT2 |
| CDC20 | Cell division cycle 20 | CDC20 |
| NEK2 | NIMA-related kinase 2 | NEK2 |
| CENPF | Centromere protein F | CENPF |
| KIF14 | Kinesin family member 14 | KIF14 |
| PPP1R3C | Protein phosphatase 1 regulatory subunit 3C | PPP1R3C |
| CEP55 | Centrosomal protein 55 | CEP55 |
| KIF2C | Kinesin family member 2C | KIF2C |
| SORBS2 | Sorbin and SH3 domain containing 2 | SORBS2 |
| RFC4 | Replication factor C subunit 4 | RFC4 |
| FEN1 | Flap endonuclease 1 | FEN1 |
| BUB1 | Budding uninhibited by benzimidazoles 1 | BUB1 |
| CDK1 | Cyclin-dependent kinase 1 | CDK1 |
| CKS1B | Cyclin-dependent kinase subunit 1B | CKS1B |
| PRC1 | Protein regulator of cytokinesis 1 | PRC1 |

Table S3. Differential expression analysis of 26 candidate genes in TCGA-GTEx datasets.

| Gene | Log_2_FC | adjusted P value |
| --- | --- | --- |
| ASPM | 5.31 | <0.001 |
| MCM2 | 3.08 | <0.001 |
| MCM6 | 1.52 | <0.001 |
| AURKA | 3.55 | <0.001 |
| TPX2 | 5.18 | <0.001 |
| BIRC5 | 4.27 | <0.001 |
| KIF4A | 4.20 | <0.001 |
| CDKN3 | 3.30 | <0.001 |
| RAD51AP1 | 3.49 | <0.001 |
| GMNN | 1.46 | <0.001 |
| TTK | 4.82 | <0.001 |
| ECT2 | 3.63 | <0.001 |
| CDC20 | 4.34 | <0.001 |
| NEK2 | 5.46 | <0.001 |
| CENPF | 4.93 | <0.001 |
| KIF14 | 5.11 | <0.001 |
| PPP1R3C | -4.32 | <0.001 |
| CEP55 | 5.06 | <0.001 |
| KIF2C | 3.48 | <0.001 |
| SORBS2 | -2.71 | <0.001 |
| RFC4 | 1.23 | <0.001 |
| FEN1 | 1.91 | <0.001 |
| BUB1 | 4.86 | <0.001 |
| CDK1 | 4.14 | <0.001 |
| CKS1B | 2.14 | <0.001 |
| PRC1 | 2.57 | <0.001 |

Table S4. Search strategy and inclusion/exclusion criteria for literature on immunohistochemistry of 26 candidate genes

| Category | Details |
| --- | --- |
| Search strategy | The search was conducted up to June 30, 2024, following PRISMA guidelines. Two databases were utilized: PubMed and Web of Science (WOS). |
|  | Keywords Used in PubMed (e.g. ECT2): ((ECT2) OR (Epithelial Cell Transforming 2)) AND ((Esophageal cancer) OR (Esophageal tumor) OR (Esophageal carcinoma)) |
|  | Keywords Used in WOS (e.g. ECT2): (TS=(ECT2) OR TS=(Epithelial Cell Transforming 2)) AND (TS=(Esophageal cancer) OR TS=(Esophageal tumor) OR TS=(Esophageal carcinoma)) |
| Inclusion criteria | - Published in English |
|  | - Focused on esophageal squamous carcinoma |
|  | - compared protein expression in esophageal squamous carcinoma and adjacent normal tissues or compared positivity rates between the two groups through immunohistochemistry |
|  | - sample size in ESCC group ≥ 10  - sample size in normal group ≥ 10 |
| Exclusion criteria | - Reviews |
|  | - Conference abstracts |
|  | - animal studies |
|  | - Studies with sample sizes < 10 in either the ESCC or normal group |

ESCC Esophageal squamous cell carcinoma.

Table S5. Basic information of studies included in literature review

| Proteins | Studies | Year | ESCC samples (n) | Control samples (n) | Estimate | *P* |
| --- | --- | --- | --- | --- | --- | --- |
| KIF4A | Wang L ^[1]^ | 2021 | 65 | 65 | positive cell percentage | <0.01 |
|  | Li Y ^[2]^ | 2021 | 60 | 60 | immunoreactive score | <0.05 |
|  | Zhang M ^[3]^ | 2022 | 50 | 50 | immunoreactive score | <0.01 |
| CDKN3 | Wang W ^[4]^ | 2021 | 184 | 50 | immunoreactive score | <0.001 |
| SURV | Yan L ^[5]^ | 2020 | 93 | 93 | staining index | <0.01 |
|  | Li C ^[6]^ | 2012 | 50 | 50 | protein positive rate | <0.001 |
|  | Gao Y ^[7]^ | 2023 | 118 | 118 | immunoreactive score | <0.001 |
| ECT2 | Zheng YQ ^[8]^ | 2024 | 20 | 18 | immunoreactive score | <0.001 |
| AURKA | Hsu PK ^[9]^ | 2014 | 97 | 97 | immunoreactive score | — |
|  | Du RJ ^[10]^ | 2023 | 94 | 94 | protein expression level | <0.001 |
|  | Mi Y^[11]^ | 2024 | 10 | 10 | protein expression level | 0.007 |
| CKS1B | Wang JJ ^[12]^ | 2013 | 56 | 56 | protein expression level | <0.05 |
|  | Wang XC ^[13]^ | 2012 | 140 | 140 | positive cell percentage | — |
| TPX2 | Hsu PK ^[9]^ | 2014 | 97 | 97 | immunoreactive score | — |
|  | Liu HC ^[14]^ | 2013 | 62 | 62 | protein positive rate | <0.01 |
| CEP55 | Yan SM ^[15]^ | 2021 | 241 | 241 | immunoreactive score | <0.001 |
|  | Jiang WP ^[16]^ | 2017 | 110 | 110 | immunoreactive score | <0.001 |
| NEK2 | Su W ^[17]^ | 2022 | 103 | 103 | immunoreactive score | <0.001 |
|  | Gu SR ^[18]^ | 2024 | 62 | 62 | immunoreactive score | <0.05 |
| KIF2C | Duan H ^[19]^ | 2016 | 415 | 40 | protein positive rate | — |
| MCM6 | Li X ^[20]^ | 2020 | 68 | 30 | protein expression level | <0.001 |

Reference

1. Wang L, Liu G, Bolor-Erdene E, Li Q, Mei Y, Zhou L. Identification of KIF4A as a prognostic biomarker for esophageal squamous cell carcinoma. Aging (Albany NY). 2021;13(21):24050-24070.

2. Li Y, Zhu X, Yang M, Wang Y, Li J, Fang J, et al. YAP/TEAD4-induced KIF4A contributes to the progression and worse prognosis of esophageal squamous cell carcinoma. Mol Carcinog. 2021;60(7):440-454.

3. Zhang M, Ren Z, Wang X, Liu C, Zheng Z, Zhao J, et al. Aspirin Exerts Its Antitumor Effect in Esophageal Squamous Cell Carcinoma by Downregulating the Expression of ATAD2 and KIF4A. Anal Cell Pathol (Amst). 2022;2022:7005328.

4. Wang W, Liao K, Guo HC, Zhou S, Yu R, Liu Y, et al. Integrated transcriptomics explored the cancer-promoting genes CDKN3 in esophageal squamous cell cancer. J Cardiothorac Surg. 2021;16(1):148.

5. Yan L, Zhao Q, Liu L, Jin N, Wang S, Zhan X. Expression of SIRT1 and survivin correlates with poor prognosis in esophageal squamous cell carcinoma. Medicine (Baltimore). 2020;99(34):e21645.

6. Li C, Yan Y, Ji W, Bao L, Qian H, Chen L, et al. OCT4 positively regulates Survivin expression to promote cancer cell proliferation and leads to poor prognosis in esophageal squamous cell carcinoma. PLoS One. 2012;7(11):e49693.

7. Gao Y, Wan L, Li M, Wang B, Ma Y. NRF2/HO-1 axis, BIRC5, and TP53 expression in ESCC and its correlation with clinical pathological characteristics and prognosis. Int J Biol Markers. 2023;38(3-4):174-184.

8. Zheng YQ, Huang HH, Chen SX, Xu XE, Li ZM, Li YH, et al. Discovery and validation of combined biomarkers for the diagnosis of esophageal intraepithelial neoplasia and esophageal squamous cell carcinoma. J Proteomics. 2024;304:105233.

9. Hsu PK, Chen HY, Yeh YC, Yen CC, Wu YC, Hsu CP, et al. TPX2 expression is associated with cell proliferation and patient outcome in esophageal squamous cell carcinoma. J Gastroenterol. 2014;49(8):1231-1240.

10. Du R, Li K, Zhou Z, Huang Y, Guo K, Zhang H, et al. Bioinformatics and experimental validation of an AURKA/TPX2 axis as a potential target in esophageal squamous cell carcinoma. Oncol Rep. 2023;49(6).

11. Mi Y, Chen L, Wang C, Miao Y, Song C, Su J, et al. AURKA knockdown inhibits esophageal squamous cell carcinoma progression through ferroptosis. Heliyon. 2024;10(7):e28365.

12. Wang JJ, Fang ZX, Ye HM, You P, Cai MJ, Duan HB, et al. Clinical significance of overexpressed cyclin-dependent kinase subunits 1 and 2 in esophageal carcinoma. Dis Esophagus. 2013;26(7):729-736.

13. Wang XC, Tian LL, Tian J, Li D, Wang Y, Wu H, et al. Overexpression of Cks1 increases the radiotherapy resistance of esophageal squamous cell carcinoma. J Radiat Res. 2012;53(1):72-78.

14. Liu HC, Zhang Y, Wang XL, Qin WS, Liu YH, Zhang L, et al. Upregulation of the TPX2 gene is associated with enhanced tumor malignance of esophageal squamous cell carcinoma. Biomed Pharmacother. 2013;67(8):751-755.

15. Yan SM, Liu L, Gu WY, Huang LY, Yang Y, Huang YH, et al. CEP55 Positively Affects Tumorigenesis of Esophageal Squamous Cell Carcinoma and Is Correlated with Poor Prognosis. J Oncol. 2021;2021:8890715.

16. Jiang W, Wang Z, Jia Y. CEP55 overexpression predicts poor prognosis in patients with locally advanced esophageal squamous cell carcinoma. Oncol Lett. 2017;13(1):236-242.

17. Su W, Hu H, Ding Q, Wang M, Zhu Y, Zhang Z, et al. NEK2 promotes the migration and proliferation of ESCC via stabilization of YAP1 by phosphorylation at Thr-143. Cell Commun Signal. 2022;20(1):87.

18. Gu S, Yasen Y, Wang M, Huang B, Zhou Y, Wang W. NEK2 promotes the migration, invasion, proliferation of ESCC and mediates ESCC immunotherapy. Heliyon. 2024;10(9):e29682.

19. Duan H, Zhang X, Wang FX, Cai MY, Ma GW, Yang H, et al. KIF-2C expression is correlated with poor prognosis of operable esophageal squamous cell carcinoma male patients. Oncotarget. 2016;7(49):80493-80507.

20. Li X, Ren Z, Xiong C, Geng J, Li Y, Liu C, et al. Minichromosome maintenance 6 complex component identified by bioinformatics analysis and experimental validation in esophageal squamous cell carcinoma. Oncol Rep. 2020;44(3):987-1002.

Table S6. Diagnostic performance of the support vector machine model in different clinical subgroups.

| Group | AUC(95%CI) | Sen(%) | Spe(%) | YI | +LR | -LR | Acc(%) | *P* for Delong |
| --- | --- | --- | --- | --- | --- | --- | --- | --- |
| Age, year | |  |  |  |  |  |  | 0.374 |
| <65 | 0.826(0.753-0.899) | 54.84 | 85.48 | 0.40 | 3.78 | 0.53 | 70.16 |  |
| ≥65 | 0.784(0.728-0.841) | 53.60 | 85.60 | 0.39 | 3.72 | 0.54 | 69.60 |  |
| Gender |  |  |  |  |  |  |  | 0.078 |
| Male | 0.827(0.777-0.876) | 61.54 | 85.38 | 0.47 | 4.21 | 0.45 | 73.46 |  |
| Female | 0.732(0.639-0.824) | 42.11 | 87.72 | 0.30 | 3.43 | 0.66 | 64.91 |  |
| TNM stage | |  |  |  |  |  |  | 0.469 |
| I-II | 0.768(0.704-0.832) | 55.38 | 85.03 | 0.40 | 3.70 | 0.52 | 77.38 |  |
| III-IV | 0.804(0.731-0.877) | 51.72 | 86.63 | 0.38 | 3.87 | 0.56 | 81.94 |  |
| Differentiation | |  |  |  |  |  |  | 0.748 |
| Low and Moderate | 0.807(0.744-0.869) | 56.86 | 88.77 | 0.46 | 5.06 | 0.49 | 81.93 |  |
| High | 0.791(0.720-0.862) | 55.81 | 85.03 | 0.41 | 3.73 | 0.52 | 79.57 |  |
| Site |  |  |  |  |  |  |  |  |
| Upper | 0.798(0.689-0.908) | 63.64 | 85.03 | 0.49 | 4.25 | 0.43 | 82.78 | 0.973^a^ |
| Middle | 0.796(0.740-0.852) | 52.11 | 86.10 | 0.38 | 3.75 | 0.56 | 76.74 | 0.464^b^ |
| Lower | 0.831(0.757-0.905) | 65.52 | 90.91 | 0.56 | 7.21 | 0.38 | 87.50 | 0.629^c^ |
| Lymphatic metastasis | |  |  |  |  |  |  | 0.272 |
| Yes | 0.813(0.747-0.879) | 58.14 | 86.10 | 0.44 | 4.18 | 0.49 | 80.87 |  |
| No | 0.761(0.695-0.826) | 53.33 | 85.03 | 0.38 | 3.56 | 0.55 | 77.33 |  |
| Distant metastasis | |  |  |  |  |  |  | 0.666 |
| Yes | 0.803(0.706-0.901) | 53.33 | 86.63 | 0.40 | 3.99 | 0.54 | 84.16 |  |
| No | 0.778(0.718-0.838) | 54.17 | 85.03 | 0.39 | 3.62 | 0.54 | 76.45 |  |

AUC area under the ROC curve, 95%CI 95% confidence interval, Sen sensitivity, Spe specificity, Acc Accuracy, YI Youden index, +LR positive likelihood ratio, -LR negative likelihood ratio. ^a^ Delong test for upper group VS middle group; ^b^ Delong test for lower group VS middle group; ^c^ Delong test for upper group VS lower group.


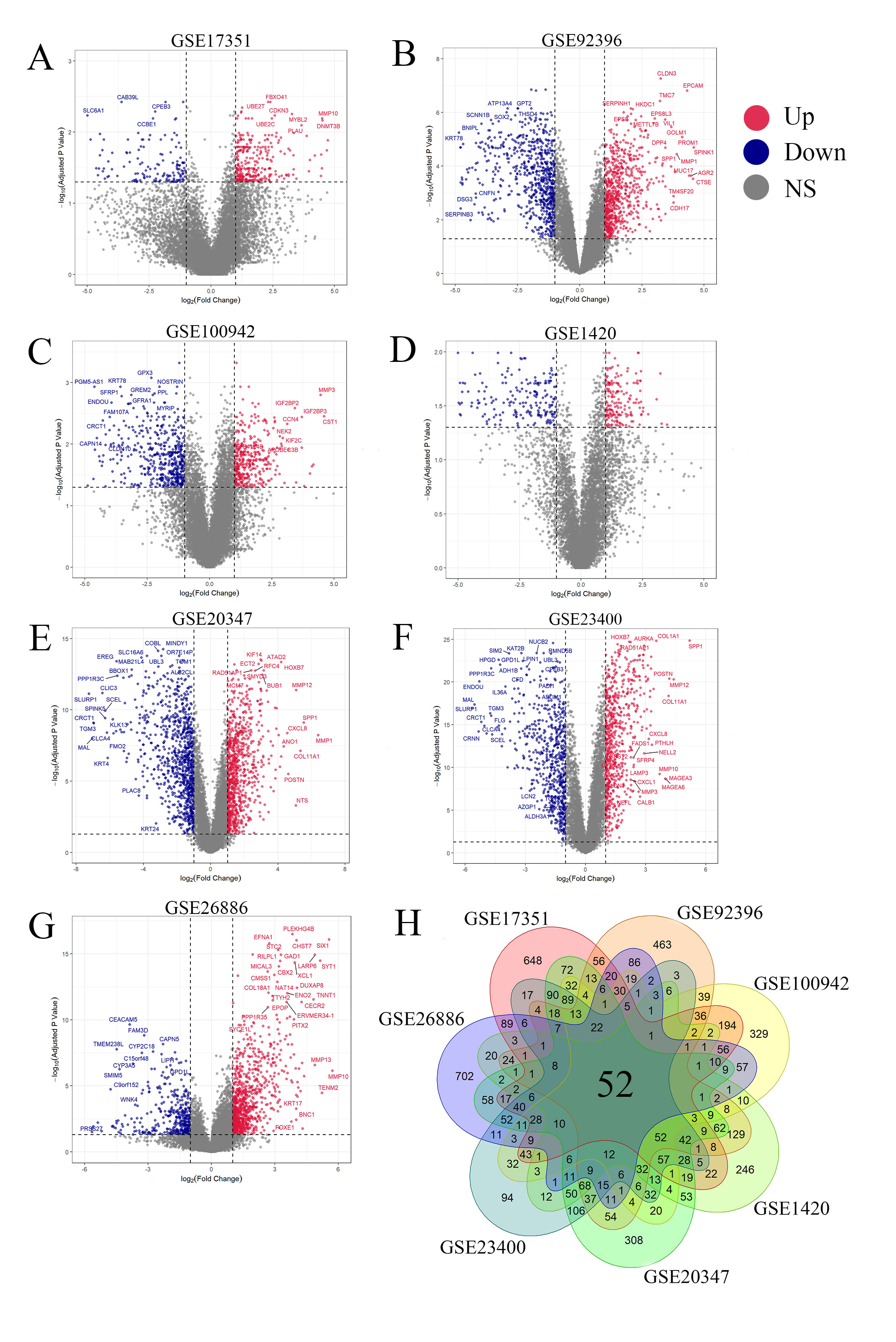


Figure S1. Volcano plots for differential expression analysis of the 7 GEO datasets and Venn diagrams for the intersection of differentially expressed genes

Note: (A-G) Limma differential expression analysis was performed for each GEO dataset, with |Log_2_FC|>1 as the threshold, and volcano maps of differentially expressed genes were screened. (H) The intersection of differentially expressed genes from 7 datasets yielded 52 common differentially expressed genes. UP: significantly up regulated; Down: significantly down; NS: No significant difference


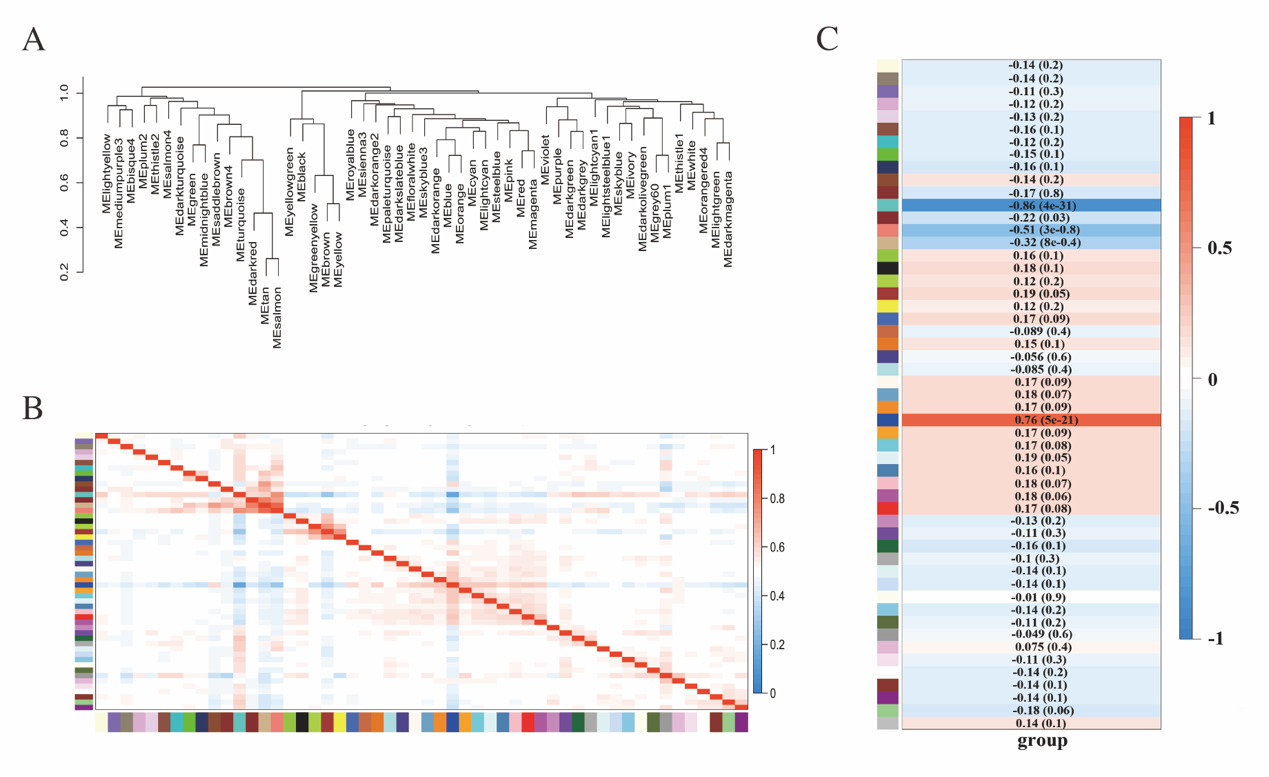


Figure S2. WGCNA analysis results.

Note: (A) Hierarchical clustering dendrogram of genes, (B) module-trait correlation heatmap, and (C) module eigengene-trait correlation heatmap.


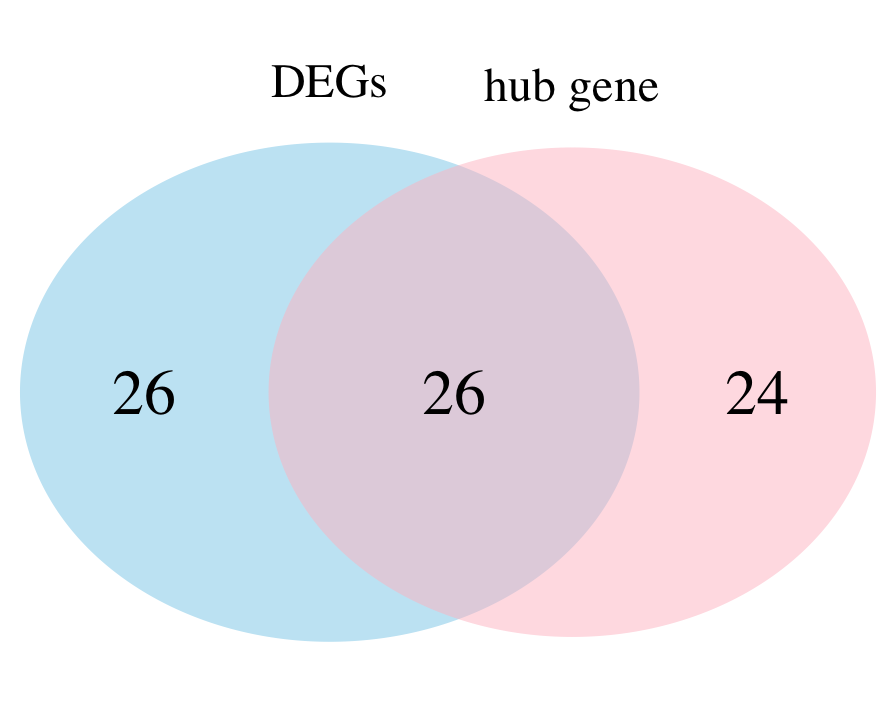


Figure S3. Venn diagram of hub genes and DEGs. DEGs: differentially expressed genes.


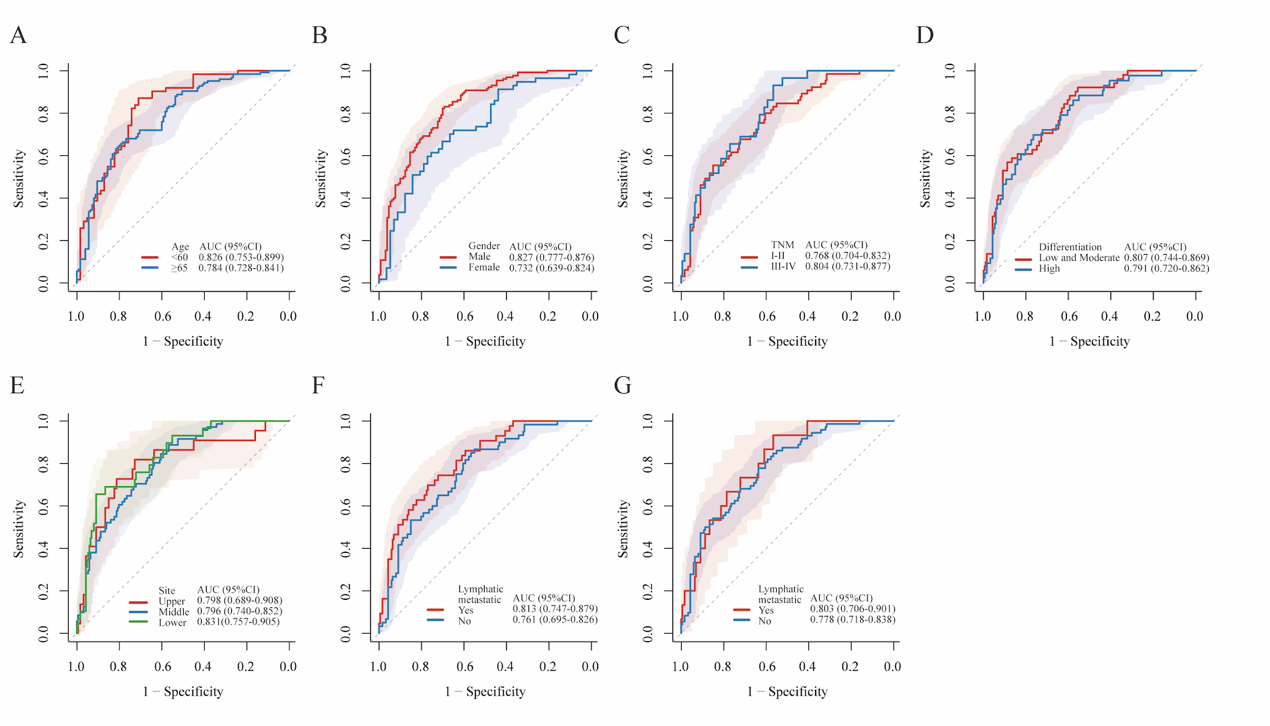


Figure S4. ROC curves of the support vector machine model in various clinical subgroups. AUC area under the ROC curve; CI confidence interval.
